# The TIMEBASE Study: IdenTifying dIgital bioMarkers of illnEss activity in BipolAr diSordEr. Preliminary results

**DOI:** 10.1192/j.eurpsy.2022.575

**Published:** 2022-09-01

**Authors:** G. Anmella, A. Mas, I. Pacchiarotti, T. Fernández, A. Bastidas, I. Agasi, M. Garriga, N. Verdolini, N. Arbelo, D. Nicolás, V. Ruiz, M. Valentí, A. Murru, E. Vieta, A. Solanes, F. Corponi, B. Li, D. Hidalgo-Mazzei

**Affiliations:** 1 Hospital Clínic de Barcelona, Department Of Psychiatry And Psychology, Barcelona, Spain; 2 Hospital Clínic de Barcelona, Psychiatry And Psychology, Barcelona, Spain; 3 Hospital Clínic de Barcelona, Psychiatry, Barcelona, Spain; 4 University of Barcelona, Bipolar And Depressive Disorders Unit, Institute Of Neuroscience, Hospital Clinic, Idibaps, Cibersam, Barcelona, Spain; 5 Hospital Clínic Barcelona, Psychiatry, Barcelona, Spain; 6 Hospital Clínic de Barcelona, Department Of Internal Medicine Psychology, Barcelona, Spain; 7 Hospital Clinic of Barcelona, University of Barcelona, IDIBAPS, CIBERSAM, Barcelona Bipolar Disorders Program, Neuroscience Institute, Barcelona, Spain; 8 Hospital Clínic de Barcelona, Bipolar And Depressive Disorders Unit, Institute Of Neuroscience, Barcelona, Spain; 9 Hospital Clinic, Psychiatry And Psychology, Barcelona, Spain; 10 IDIBAPS, Imaging Of Mood-and Anxiety-related Disorders, Barcelona, Spain; 11 University of Edinburgh, School Of Informatics, Edimburgh, United Kingdom

**Keywords:** bipolar disorder, wearable, digital biomarker

## Abstract

**Introduction:**

Mood episodes in bipolar disorder (BD) are still identified with subjective retrospective reports and scales. Digital biomarkers, such as actigraphy, heart rate variability, or ElectroDermal activity (EDA) have demonstrated their potential to objectively capture illness activity.

**Objectives:**

To identify physiological digital signatures of illness activity during acute episodes of BD compared to euthymia and healthy controls (HC) using a novel wearable device (Empatica´s E4).

**Methods:**

A pragmatic exploratory study. The sample will include 3 independent groups totalizing 60 individuals: 36 BD inpatients admitted due to severe acute episodes of mania (N=12), depression (N=12), and mixed features (N=12), will wear the E4-device at four timepoints: the acute phase (T0), treatment response (T1), symptoms remission (T2) and during euthymia (T3; outpatient follow-up). 12 BD euthymic outpatients and 12 HC will be asked to wear the E4-device once. Data pre-processing included average downsampling, channel time-alignment in 2D segments, 3D-array stacking of segments, and random shuffling for training/validation sets. Finally, machine learning algorithms will be applied.

**Results:**

A total of 10 patients and 5 HC have been recruited so far. The preliminary results follow the first differences between the physiological digital biomarkers between manic and depressive episodes. 3 fully connected layers with 32 hidden units, ectified linear activation function (ReLU) activation, 25% dropout rate, significantly differentiated a manic from a depressive episode at different timepoints (T0, T1, T2).

**Conclusions:**

New wearables technologies might provide objective decision-support parameters based on digital signatures of symptoms that would allow tailored treatments and early identification of symptoms.

**Disclosure:**

No significant relationships.

